# Apolipoprotein C3 Is Downregulated in Patients With Inflammatory Bowel Disease

**DOI:** 10.14309/ctg.0000000000000500

**Published:** 2022-05-18

**Authors:** Alejandro Hernández-Camba, Marta Carrillo-Palau, Laura Ramos, Laura de Armas-Rillo, Milagros Vela, Laura Arranz, Miguel Á. González-Gay, Iván Ferraz-Amaro

**Affiliations:** 1 Division of Gastroenterology, Hospital Universitario de Nuestra Señora de la Candelaria, Tenerife, Spain;; 2 Division of Gastroenterology, Hospital Universitario de Canarias, Tenerife, Spain;; 3 Division of Health Sciences, Universidad Europea de Canarias, Tenerife, Spain;; 4 Division of Rheumatology, Hospital Universitario Marqués de Valdecilla, Universidad de Cantabria, Santander, Spain;; 5 Epidemiology, Genetics and Atherosclerosis Research Group on Systemic Inflammatory Diseases, Hospital Universitario Marqués de Valdecilla, IDIVAL, Santander, Spain;; 6 Cardiovascular Pathophysiology and Genomics Research Unit, School of Physiology, Faculty of Health Sciences, University of the Witwatersrand, Johannesburg, South Africa;; 7 Division of Rheumatology, Hospital Universitario de Canarias, Tenerife, Spain.

## Abstract

Inflammatory bowel disease (IBD) has been associated with an abnormal lipid profile. Apolipoprotein C-III (ApoC3) is a key molecule of triglyceride metabolism that is known to be related to inflammation and cardiovascular disease. In this study, we aim to study whether ApoC3 serum levels differ between patients with IBD and controls and whether the hypothetical disturbance of ApoC3 can be explained by IBD characteristics.

**METHODS:**

This is a cross-sectional study that included 405 individuals, 197 patients with IBD and 208 age-matched and sex-matched controls. ApoC3 and standard lipid profiles were assessed in patients and controls. A multivariable analysis was performed to analyze whether ApoC3 serum levels were altered in IBD and to study their relationship to IBD characteristics.

**RESULTS:**

After fully multivariable analysis including cardiovascular risk factors, use of statins, and changes in lipid profile caused by the disease itself, patients with IBD showed significant lower serum levels of ApoC3 (beta coef. −1.6 [95% confidence interval −2.5 to −0.7] mg/dL, *P* = 0.001). Despite this, inflammatory markers, disease phenotypes, or disease activity of IBD was not found to be responsible for this downregulation.

**DISCUSSION:**

Apolipoprotein C3 is downregulated in patients with IBD.

## INTRODUCTION

Inflammatory bowel disease (IBD), which includes Crohn's disease (CD) and ulcerative colitis (UC), is characterized by mucosal immune system dysregulation leading to an activation of intestinal mucosal inflammation. IBD has been associated with an abnormal lipid profile, and it is believed that the inflammation present in the disease is responsible for this modification in lipid molecules (1,2). Moreover, although lipoprotein alterations of patients with IBD have not been consistently characterized, it is known that IBD shares modifications in lipid molecules that are present in inflammatory states consisting in lower both total cholesterol and low-density lipoprotein (LDL) cholesterol (3). This is of importance because, in a recent report, modifications of the lipid profile were independently associated with hospitalizations (low cholesterol) and IBD surgeries (low cholesterol and high triglycerides) (2).

Apolipoprotein C-III (ApoC3) is a 79 amino acid peptide synthesized in the liver and intestine that is found on the surface of triglycerides and inhibits their lipolysis by lipoprotein lipase, thereby increasing plasma levels of atherogenic triglycerides, including remnant cholesterol. Consequently, high levels of ApoC3 have been found to be associated with elevated triglycerides levels. Moreover, ApoC3 has been related to an increased risk of atherosclerotic cardiovascular (CV) disease (4). It is believed that this occurs by at least 3 mechanisms linked to ApoC3: inhibiting the lipolysis of triglyceride; augmenting arterial inflammation through effects on both peripheral monocytes and endothelial cells; and interfering with normal nitric oxide function in endothelial cells, perhaps through interfering with insulin signaling and thus causing endothelial dysfunction. This has been supported by the fact that overexpression of the ApoC3 gene during a lifetime has been associated with increased risk of coronary artery disease, whereas reduction of plasma levels of ApoC3 reduces the risk of developing coronary atherosclerosis (5). Moreover, subjects who have mutations of the ApoC3 gene causing loss of function have 40% reduction in risk of developing coronary artery disease (6,7). Interestingly, ApoC3 has also been related to inflammation (8). In this sense, ApoC3 has been described to induce inflammation and organ damage by alternative inflammasome activation (8), and ApoC3 serum levels were associated with individual metabolic syndrome risk factors such as diabetes and inflammatory markers (9).

Because ApoC3 is a component of lipid profile and has been linked to inflammation and CV disease, in this work, we aim to study how ApoC3 is expressed in patients with IBD compared with controls. In a further step, we additionally set out to analyze how ApoC3 is related to features of IBD including disease activity.

## METHODS

### Study participants

This was a cross-sectional study that included 197 patients with IBD and 208 sex-matched and age-matched controls. All patients with IBD were older than 18 years; had an IBD diagnosis of ≥1 year, based on clinical, endoscopic, and histological criteria; and were periodically followed up at IBD units of 2 tertiary hospitals. Patients or controls taking statins and systemic steroids were allowed to participate in this study. Controls were recruited by general practitioners in primary health centers. Controls with a family history of any IBD or other autoimmune disorder were excluded. Exclusion criteria for both patients and controls were established as CV disease, a glomerular filtration rate of <60 mL/min/1.73 m^2^, a history of cancer and/or any other chronic inflammatory disease, or evidence of active infection or any condition or pharmacological treatment that could influence lipids.

The study protocol was approved by the Institutional Review Committee at Hospital Universitario de Canarias and Hospital Universitario Nuestra Señora de La Candelaria, both in Spain, and all subjects provided informed written consent (approval no. CHUC_2019_103). Research conducted with human subjects was in compliance with the Helsinki Declaration.

### Data collection and laboratory assessments

Surveys in patients with IBD and controls were performed to assess CV risk factors and medication. Hypertension was defined as a systolic or diastolic blood pressure higher than 140 and 90 mmHg, respectively. Standard techniques were used to measure high-sensitivity C-reactive protein and fecal calprotectin. Disease activity in CD was assessed by the Crohn's Disease Activity Index (CDAI) and the Harvey-Bradshaw Index (HBI) (10). CDAI was broken down into asymptomatic remission (0–149 points), mildly to moderately active (150–220 points), moderately to severely active (221–450 points), and severely active to fulminant disease (451–1,100 points) categories as previously described (11). Similarly, HBI was categorized as remission (0–4 points), mildly active disease (5–7 points), moderately active disease (8–16 points) and severely active disease (17–100 points) (10). Disease activity in UC was calculated by the partial Mayo Clinic score (pMS) (12). An ELISA kit was used for the detection of ApoC3 (Elabscience Biotechnology, China). With this kit, no significant cross-reactivity or interference is observed between human ApoC3 and its analogs. Both intra-coefficients and inter-coefficients of variability are < 10% for this assay. Cholesterol, triglycerides, and HDL-cholesterol were measured using the enzymatic colorimetric assay. LDL-cholesterol was calculated using the Friedewald formula.

### Statistical analysis

Continuous variables data are expressed as mean ± SD or as median and interquartile range (IQR) for non-normally distributed variables. Univariable differences between patients and controls were assessed through Student *t*, Mann-Whitney *U* , χ^2^, or Fisher exact tests according to normal distribution or the number of subjects. Differences between patients and controls regarding their lipid profiles were assessed using multivariable linear regression analysis using controls as the reference variable (beta coefficients express the effect of IBD over controls). Confounding variables in this analysis were those with a statistical *P* value of <0.20 for those differences in traditional CV risk factors between patients and controls. To neutralize the effect of other modifications on the lipid profile, an additional multivariable analysis was constructed, adding to the model those differences in lipid-related molecules between patients and controls with a *P* value of <0.20. The relation of demographics and disease-related data to ApoC3 in patients with IBD was analyzed using multivariable linear regression analysis (ApoC3 as dependent variable). Regression linear analyses in our work were performed using the ‘enter’ method because we were interested in the effect of an independent variable on a dependent one but not in its predictive capacity. All the analyses used a 5% 2-sided significance level and were performed using SPSS software, v. 26 (IBM, Chicago, IL) and STATA software, v.17/BE (Stata Corp., College Station, TX). A *P* value of < 0.05 was considered statistically significant.

## RESULTS

### Demographic, laboratory, and disease-related data

A total of 197 IBD patients with median disease duration 12 years (IQR 8–19) and 208 age-matched and sex-matched controls with a mean ± SD age of 49 ± 10 and 50 ± 15 years, respectively, were included in this study. Demographic and disease-related characteristics of the subjects are presented in Table 1. Body mass index (27 ± 5 vs 30 ± 3 kg/m^2^, *P*=<0.001) and waist circumference (94 ± 12 vs 98 ± 7 cm, *P* < 0.001) were lower in patients with IBD than those in controls. While there were no differences in the prevalence of smoking or obesity, patients with IBD were less commonly hypertensive (18% vs 30%, *P* = 0.003) and diabetic (6% vs 14%, *P* = 0.004).

**Table 1.** Characteristics of patients with IBD and controls

	Controls n = 208	Patients with IBD n = 197	*P*
Age, yr	50 ± 15	49 ± 10	0.25
Female, n (%)	124 (59)	107 (54)	0.28
Body mass index, kg/m^2^	30 ± 3	27 ± 5	**<0.001**
Abdominal circumference, cm	98 ± 7	94 ± 12	**<0.001**
Systolic blood pressure, mmHg	125 ± 13	126 ± 19	0.45
Diastolic blood pressure, mmHg	81 ± 5	74 ± 11	<0.001
CV comorbidity			
Smoking, n (%)	45 (22)	39 (20)	0.65
Diabetes, n (%)	29 (14)	11 (6)	**0.004**
Hypertension, n (%)	63 (30)	35 (18)	**0.003**
Obesity, n (%)	57 (27)	55 (28)	0.91
Statins, n (%)	47 (23)	21 (11)	**0.001**
IBD-related data			
CD, n (%)		130 (66)	
UC, n (%)		67 (34)	
CRP, mg/L	2.0 (1.0–4.8)	1.8 (0.9–3.8)	0.30
Disease duration since diagnosis, yr	12 (8–19)		
CD-related data, n (%)			
A1 younger than 16 yr		19 (14)	
A2 between 17 and 40 yr		81 (62)	
A3 older than 40 yr		27 (21)	
L1 ileal		56 (43)	
L2 colonic		23 (18)	
L3 ileocolonic		51 (39)	
L4 isolated upper disease		11 (8)	
B1 nonstricturing and nonpenetrating		73 (56)	
B2 stricturing		46 (35)	
B3 penetrating		14 (11)	
CDAI score		39 (7–80)	
Asymptomatic remission		116 (89)	
Mildly to moderately active		10 (8)	
Moderately to severely active		3 (2)	
Severely active to fulminant disease		0 (0)	
Harvey-Bradshaw Index		2 (0–4)	
Clinical remission		106 (82)	
Mildly active disease		14 (11)	
Moderately active disease		8 (6)	
Severely active disease		1 (1)	
UC-related data, n (%)			
Proctosigmoiditis		7 (10)	
Left-sided colitis		23 (35)	
Pancolitis		34 (52)	
Partial Mayo score		1 (0–1)	
<2		52 (78)	
≥2		15 (21)	
Fecal calprotectin, mcg/g	113 (30–251)		
>150		96 (49)	
≥150		71 (36)	
Perianal disease, n (%)	23 (12)		
Previous surgery, n (%)	55 (28)		
Current prednisone, n (%)	6 (2)		
Prednisone, mg/d	8 (5–20)		
Oral mesalazine, n (%)	175 (89)		
Methotrexate, n (%)	22 (11)		
Azathioprine, n (%)	61 (31)		
Anti-TNF therapy, n (%)	58 (29)		
Ustekinumab, n (%)	8 (4)		
Vedolizumab, n (%)	5 (3)		
Tofacitinib, n (%)	4 (2)		

Significant *p* values are depicted in bold.

Data represent means ± SD or median (IQR) when data were not normally distributed.

CDAI was categorized as 0–149 points: asymptomatic remission; 150–220 points: mildly to moderately active; 221–450 points: moderately to severely active; and 451–1,100 points: severely active to fulminant disease.

HBI was categorized as 0 to 4 points: clinical remission; 5–7 points: mildly active disease; 8 to 16 points: moderately active disease; and 17–100 points: severely active disease.

CD, Crohn's disease; CDAI, Crohn's Disease Activity Index; CRP, C-reactive protein; CV, cardiovascular; HBI, Harvey-Bradshaw Index; HDL, high-density lipoprotein; IBD, inflammatory bowel disease; IQR, interquartile range; LDL, low-density lipoprotein; TNF, tumor necrosis factor; UC, ulcerative colitis.

The median disease duration was 12 years (IQR 8–19). Patients with CD had mostly the ileal and nonstricturing, nonpenetrating types. The median CDAI score was 39 (IQR 7–80) and 89% of the patients were considered to be in the asymptomatic remission category. Similarly, median HBI was 2 (IQR 0–4) and most of the patients (82%) were in the remission category of this index. Regarding UC, 52% had pancolitis, and 76% of the patients had a pMS inferior to 2 points. Additional information regarding disease-related data is given in Table 1.

### Multivariable analysis of the differences in lipid profiles and apolipoprotein C3 between patients with IBD and controls

In this analysis, controls are considered the reference variable and, therefore, differences are shown as the effect of having IBD against being a control. In this sense, although total cholesterol, triglycerides, LDL-cholesterol, non-high-density lipoprotein (HDL) cholesterol, lipoprotein (a), and apolipoprotein B did not differ, some differences were observed in the lipid profile between IBD controls and patients. In the univariable analysis (Table 2), HDL-cholesterol (57 ± 18 mg/dL vs 51 ± 14 mg/dL, *P* = 0.001) and ApoB:ApoA1 ratio (0.69 ± 0.22 vs 0.62 ± 0.18, *P* = 0.001) were found to be significantly higher in patients with IBD compared with controls. Contrarily, LDL:HDL ratio, apolipoprotein A1, and atherogenic index were significantly lower in patients with IBD than those in controls in the univariable analysis. In addition, circulating ApoC3 disclosed inferior serum levels in patients compared with controls (3.5 [IQR 2.8–4.4] vs 4.1 [IQR 2.5–6.9] mg/dL, *P* < 0.001).

**Table 2.** Multivariable analysis of the differences in lipid profile and apolipoprotein C3 between patients with IBD and controls

	Controls (n = 208)	Patients with IBD (n = 197), *P*	Univariable model	Model #1 beta coef. (95% CI), *P*	Model #2 beta coef. (95% CI), *P*
Lipid profile					
Cholesterol, mg/dL	198 ± 45	203 ± 49	0.35		
Triglycerides, mg/dL	144 ± 70	151 ± 89	0.38		
HDL cholesterol, mg/dL	51 ± 14	57 ± 18	**0.001**	3 (0 to 6), 0.070	**8 (7 to 10), <0.001**
LDL cholesterol, mg/dL	118 ± 37	116 ± 40	0.56		
LDL:HDL cholesterol ratio	2.42 ± 0.88	2.18 ± 0.86	**0.005**	**−0.20 (−0.39 to −0.02), 0.029**	—
Non-HDL cholesterol, mg/dL	147 ± 40	146 ± 43	0.81		
Lipoprotein (A), mg/dL	38 (14–103)	26 (8–88)	0.37		
Apolipoprotein A1, mg/dL	173 ± 39	162 ± 37	**0.003**	**−13 (−21 to −6), 0.001**	**−21 (−25 to −17), <0.001**
Apolipoprotein B, mg/dL	105 ± 29	108 ± 32	0.29		
ApoB:ApoA1 ratio	0.62 ± 0.18	0.69 ± 0.22	**0.001**	**0.08 (0.04 to 0.12), <0.001**	—
Atherogenic index	4.05 ± 1.11	3.80 ± 1.17	**0.025**	−0.09 (−0.33 to 0.14), 0.43	
Apolipoprotein C3, mg/dL	4.1 (2.5–6.9)	3.5 (2.8–4.4)	**<0.001**	**−1.5 (−2.3 to −0.6), <0.001**	**−1.6 (−2.5 to −0.7), 0.001**

In the linear regression analysis, controls are considered the reference variable. Beta coefficients express the effect of IBD against controls.

Data represent means ± SD or median (interquartile range) when data were not normally distributed.

Model #1: adjusted for body mass index, abdominal circumference, hypertension, diabetes, and statins (variables with a *P* value < 20 difference between patients and controls).

Model #2: adjusted for model #1 + rest of lipid molecules (with a *P* value of < 0.20 in the univariate analysis) other than the one that is compared. Because of collinearity, the LDL:HDL and ApoB:ApoA1 ratios were excluded from the multivariable analyses in model 2.

Significant *P* values are depicted in bold.

Apo, apolipoprotein; CI, confidence interval; HDL, high-density lipoprotein; IBD, inflammatory bowel disease; LDL, low-density lipoprotein.

In the fully adjustment model (Model 1 in Table 2), most of these differences between the 2 populations were maintained with some exceptions. Hence, the LDL:HDL ratio and apolipoprotein A1 remain significantly lower and the ApoB:ApoA1 ratio was found to be higher in patients with IBD. The differences between HDL-cholesterol serum levels and atherogenic index became not significant. Remarkably, ApoC3 persisted significantly downregulated in patients with IBD compared with controls (beta coef. −1.5 [95% confidence interval [[CI]] −2.3 to −0.6] mg/dL, <0.001).

Because lipid-related molecules are interrelated, we performed a multivariable analysis adjusting for demographics and CV risk factors plus all the lipid-related molecules (they share metabolic pathways and it is not easy to separate the effect of one from the others) that were found to be different between patients and controls in the univariable analysis (Model 2 in Table 2). Because of collinearity, lipid molecules derived from a formula were excluded from the regression models (LDL-cholesterol, LDL:HDL ratio, non-HDL cholesterol, ApoB:ApoA1, and atherogenic index). In this final multivariable model, ApoC3 was found to stay lower in patients with IBD compared with that in controls (beta coef. −1.6 [95% CI -2.5 to −0.7] mg/dL, <0.001).

### Disease-related data relation with apolipoprotein C3

The association of demographics and disease-related data with ApoC3 (as dependent variable) is presented in Table 3. In this sense, some classical CV risk factors such as age and the presence of hypertension were significantly and positively related to higher serum levels of ApoC3. Besides, the use of statins was significantly associated with superior circulating ApoC3 (Table 3). Concerning disease-related data, disease duration and phenotypes, inflammation markers such as C-reactive protein and fecal calprotectin, and the use of different treatments were not associated with ApoC3. Patients with CD in the HBI moderately active disease category disclosed higher levels of ApoC3 compared with those in clinical remission after multivariable analysis (beta coef. 1.3 [95% CI 0.2–2.4], *P* = 0.022). However, this relation was not found for other disease activity scores such as CDAI or partial Mayo (Table 3).

**Table 3.** Relation of demographics and disease-related data to ApoC3

	ApoC3, mg/dL			
	Beta coef. (95% CI), *P*			
	Univariable		Adjusted	
Age, yr	**0.06 (0.03 to 0.10)**	**0.002**		
Female	−0.5 (−1.1 to 0.1)	0.097		
Body mass index, kg/m^2^	−0.01 (−0.07 to 0.05)	0.68		
Abdominal circumference, cm	0.00 (−0.02 to 0.03)	0.92		
Systolic blood pressure, mmHg	0.01 (0.00 to 0.03)	0.11		
Diastolic blood pressure, mmHg	0.01 (−0.02 to 0.04)	0.51		
CV comorbidity				
Smoking	−0.1 (−1.0 to 0.6)	0.71		
Diabetes	0.2 (−1.2 to 1.5)	0.81		
Hypertension	**1.1 (0.3 to 1.9)**	**0.006**		
Obesity	−0.2 (−0.8 to 0.5)	0.63		
Statins	**1.6 (0.7 to 2.6)**	**0.001**		
IBD-related data				
CD	0.4 (−0.3 to 1.0)	0.28		
UC				
CRP, mg/L	0.03 (−0.04 to 0.10)	0.41		
Disease duration since diagnosis, yr	0.02 (−0.01 to 0.05)	0.23		
CD-related data				
A1 younger than 16 yr	−0.2 (−1.0 to 0.7)	0.68		
A2 between 17 and 40 yr	−0.2 (−0.8 to 0.4)	0.44		
A3 older than 40 yr	0.7 (−0.1 to 1.4)	0.069	0.2 (−0.6 to 0.9)	0.68
L1 ileal	−0.2 (−0.8 to 0.4)	0.52		
L2 colonic	−0.4 (−1.2 to 0.4)	0.31		
L3 ileocolonic	0.5 (−0.1 to 1.1)	0.077	0.5 (−0.1 to 1.0)	0.11
L4 isolated upper disease	−0.8 (−1.8 to 0.3)	0.15		
B1 nonstricturing and nonpenetrating	−0.1 (−0.6 to 0.5)	0.82		
B2 stricturing	−0.1 (−0.8 to 0.5)	0.66		
B3 penetrating	0.1 (−0.8 to 1.1)	0.82		
CDAI score	0.0 (0.0 to 0.0)	0.70		
Asymptomatic remission	ref.		ref.	
Mildly to moderately active	−0.8 (−1.9 to 0.3)	0.14	−0.4 (−1.5 to 0.7)	0.44
Moderately to severely active	0.4 (−1.5 to 2.3)	0.68	0.7 (−1.2 to 2.6)	0.44
Harvey-Bradshaw Index	0.0 (−0.1 to 0.1)	0.40		
Clinical remission	ref.		ref.	
Mildly active disease	−0.5 (−1.4 to 0.5)	0.33	−0.3 (−1.2 to 0.6)	0.53
Moderately active disease	**1.3 (0.1 to 2.4)**	**0.028**	**1.3 (0.2 to 2.4)**	**0.022**
Ulcerative colitis-related data				
Proctosigmoiditis	−0.8 (3.1 to 1.5)	0.49		
Left-sided colitis	−0.2 (−1.7 to 1.3)	0.77		
Pancolitis	0.3 (−1.1 to 1.8)	0.63		
Partial Mayo score	0.1 (−0.3 to 0.6)	0.61		
<2	ref.			
≥2	0.5 (−1.2 to 2.2)	0.54		
Fecal calprotectin, mcg/g	0.00 (0.00 to 0.00)	0.29		
<150	ref.			
≥150	0.4 (−0.3 to 1.0)	0.31		
Perianal disease	0.0 (−1.0 to 1.0)	0.99		
Previous surgery	0.1 (−0.6 to 0.7)	0.85		
Current prednisone	−1.0 (−2.8 to 0.7)	0.26		
Prednisone, mg/d	0.0 (−0.1 to 0.1)	0.83		
Oral mesalazine	**0.7 (0.1 to 1.3)**	**0.024**	0.11	
Methotrexate	**1.3 (0.3 to 2.2)**	**0.008**	0.059	
Azathioprine	−0.2 (−0.8 to 0.5)	0.62		
Anti-TNF therapy	0.4 (−0.3 to 1.1)	0.22		
Ustekinumab	0.2 (−1.4 to 1.7)	0.84		
Vedolizumab	−0.3 (2.2 to 1.7)	0.80		
Tofacitinib	−0.8 (−3.0 to 1.3)	0.44		

ApoC3 is considered the dependent variable in this analysis. Beta coefficients express the effect of demographics and disease-related data on ApoC3. Multivariable analysis is adjusted for age, sex, hypertension, and statin use.

CDAI was categorized as 0–149 points: asymptomatic remission; 150–220 points: mildly to moderately active; 221–450 points: moderately to severely active; and 451–1,100 points: severely active to fulminant disease.

HBI was categorized as 0–4 points: clinical remission; 5–7 points: mildly active disease; 8–16 points: moderately active disease; and 17–100 points: severely active disease.

Significant *P* values are depicted in bold.

ApoC3, apolipoprotein C-III; BMI, body mass index; CD, Crohn's disease; CDAI, Crohn's Disease Activity Index; CI, confidence interval; CRP, C reactive protein; HBI, Harvey-Bradshaw Index; HDL, high-density lipoprotein; IBD, inflammatory bowel disease; LDL, low-density lipoprotein; TNF, tumor necrosis factor; UC, ulcerative colitis.

## DISCUSSION

ApoC3 expression in patients with IBD has not been explored in the literature before this study. According to our results, patients with IBD show significant inferior serum levels of this apolipoprotein compared with controls.

An apparent lipid paradox has been described in inflammatory states. For example, in the prototype of inflammatory disease, rheumatoid arthritis, active disease with high burden of inflammatory markers is usually accompanied with low levels of LDL-cholesterol, total cholesterol, and HDL-cholesterol. By contrast, decreases in inflammation may coincide with increases in serum lipid values (13,14). These paradoxical lipid profiles have also been found in other inflammatory states such as systemic lupus erythematosus (15), spondyloarthritis (16), heart failure, ischemic heart disease, cancer, or sepsis (17). However, in our study on patients with IBD, we have not found the presence of this paradoxical lipid profile. The fact that most of the patients had low activity or were in remission may have contributed to this finding. Moreover, previous studies on IBD have not been uniform in defining a specific lipid profile for this disease. For example, in a previous work of 701 patients with IBD and matched controls, low total cholesterol and high triglyceride levels were more frequent in IBD compared with healthy subjects, and this was independently associated with more severe disease (2). Contrarily, in a report of 393 patients, total cholesterol and HDL were significantly lower and LDL and triglycerides were significantly greater in male patients with IBD. In female patients with IBD, the mean values for total cholesterol, HDL, and triglycerides were significantly lower and LDL-C significantly greater compared with the female controls (1).

In our study, a positive relationship was found between ApoC3 and CD's HBI. This relation was significant after multivariable analysis. However, this association was not found with other disease activity scores or serum markers of inflammation. On the other hand, only 7 patients were in the moderately active category of this index. For this reason, we believe that this finding should be taken with caution. Moreover, the fact that only some traditional CV risk factors were associated with ApoC3, but most disease-related data were not, is noteworthy. We believe that the disease itself, by mechanisms that are not captured through the clinical manifestations or scores assessed in our work, may be responsible for the downregulation of ApoC3. Remarkably, ApoC3 downregulation was not a consequence of modifications in other lipid molecules because it remained significantly observed after multivariable analysis that included those other modifications that the disease exerted over the lipid profile.

Owing to the fact that ApoC3 was lower in patients with IBD, it might have been expected that triglycerides were also inferior; however, this was not the case. We do not have an exact explanation for this. We speculate that IBD has alternative mechanisms to downregulate ApoC3 that does not affect triglycerides or that, in IBD, triglyceride metabolism is not completely regulated by ApoC3 or has other feedback pathways.

IBD has been associated with an accelerated subclinical atherosclerosis (18) and with an increased incidence of CV events (19). Similarly, elevated levels of ApoC3 are associated with superior triglyceride levels and an increased risk of atherosclerotic CV disease (7). For this reason, our findings that ApoC3 serum levels were inferior in patients with IBD compared with controls are to some extent unexpected. We understand that these findings are in line with the fact that certain molecules of lipid metabolism, in inflammatory diseases, may be decreased for reasons that are unknown but probably related to the background inflammation that is present in these diseases.

We acknowledge the limitation that our sample can be considered small in size. However, recruiting almost 200 patients, and a similar number of controls, has allowed us to perform a full multivariable analysis adjusted for numerous confounders. We also recognize that our cross-sectional design allows us to study associations but not causality. For this reason, future studies with a prospective design are warranted to study the real role that ApoC3 has in patients with IBD and its relationship with the characteristics of the disease or its associated CV disease. Moreover, our work lacked endoscopic scores. This has not permitted us to study the relation of these scores to ApoC3 serum levels.

In conclusion, ApoC3 is independently downregulated in patients with IBD compared with matched controls. The role of ApoC3 in the increased CV disease of patients with IBD deserves further studies in the future.

## CONFLICTS OF INTEREST

**Guarantor of the article:** Iván Ferraz-Amaro, MD.

**Specific author contributions:** A.H.C., M.C.P., and L.R.P.: study design, data collection, and critical revision of the manuscript. L.R.R., data collection. I.F.A. and M.A.G.G.: data analysis and writing the first draft of the manuscript. M.A.G.G. and I.F.A.: critical revision of the manuscript.

**Financial support:** This work was supported by a grant to I.F-A. from the Spanish Ministry of Health, Subdirección General de Evaluación y Fomento de la Investigación, Plan Estatal de Investigación Científica y Técnica y de Innovación 2013–2016 and by Fondo Europeo de Desarrollo Regional—FEDER(Fondo de Investigaciones Sanitarias, FIS PI14/00394, PI17/00083, and PI20/00084).

**Potential competing interests:** The authors declare that there are no conflicts of interest. Nevertheless, Dr. Iván Ferraz-Amaro would like to acknowledge that he has received grants/research supports from Abbott, MSD, Janssen, and Roche, as well as consultation fees from company sponsored speakers bureaus associated with Abbott, Pfizer, Roche, Sanofi, Celgene, and MSD. Dr. M.A. González-Gay has received grants/research supports from AbbVie, MSD, Janssen, and Roche, as well as consultation fees/participation from company-sponsored speakers bureaus tied to AbbVie, Pfizer, Roche, Sanofi, Lilly, Celgene, and MSD.

**Study Highlights**

**WHAT IS KNOWN**

✓ Inflammatory bowel disease (IBD) has been associated with an abnormal lipid profile and with an accelerated cardiovascular (CV) disease.
✓ Apolipoprotein C3 (ApoC3) is a component of lipid profile that has been linked to inflammation and CV disease, but its expression in patients with IBD is unknown.

**WHAT IS NEW HERE**

✓ Patients with IBD show significant inferior serum levels of ApoC3 compared with controls.
✓ This downregulation was independent of classical CV risk factors, use of statins, and changes in lipid profile caused by the disease itself.
